# A novel CLCN5 frame shift mutation responsible for Dent disease 1: Case report

**DOI:** 10.3389/fped.2022.1043502

**Published:** 2022-11-14

**Authors:** Jiajia Ni, Yaju Zhu, Fujun Lin, Wenbin Guan, Jing Jin, Yufeng Li, Guimei Guo

**Affiliations:** ^1^ Department of Pediatric Nephrology, Rheumatology and Immunology, Xinhua Hospital Affiliated to Shanghai Jiaotong University School of Medicine, Shanghai, China; ^2^ Department of Nephrology, Xin Hua Hospital, School of Medicine, Shanghai Jiao Tong University, Shanghai, China; ^3^ Department of Pathology, Xin Hua Hospital, School of Medicine, Shanghai Jiao Tong University, Shanghai, China

**Keywords:** Dent disease, CLCN5 gene mutation, low molecular weight proteinuria, renal tubule disorders, X-link

## Abstract

**Background:**

Dent disease is a group of inherited X-linked recessive renal tubular disorders. This group of disorders is characterized by low molecular weight proteinuria (LMWP), nephrocalcinosis, hypercalciuria and renal failure.

**Case presentation:**

Here we report one 11-year-old Chinese boy (proband) and one 13-year-old Chinese boy who was proband's cousin, both presented with massive proteinuria. Further laboratory examinations revealed a lack of nephrocalcinosis, nor any other signs of tubular dysfunction, but only LMWP and hypercalciuria. There was no abnormality in growth, renal function or mineral density of the bones. A novel deletion (c.1448delG) in the *CLCN5* gene was identified, resulting in a frame shift mutation (p.Gly483fs). The proband's and his cousin's mothers were found to be the carrier of this mutation.

**Conclusions:**

In this study, we have found a novel frameshift mutation (c. 1448delG) at exon 11 of the *CLCN5* gene which leads to Dent disease 1, expanding the spectrum of *CLCN5* mutations.

## Introduction

Dent disease is a X-linked recessive inherited renal tubule disorders (1). Two genetic subtypes have been identified: *CLCN5*-mutated Dent disease type 1 and *OCRL*-mutated Dent disease type 2 (2). There wasn’t a clear causative gene found in about 25% Dent patients (3, 4). The chloride channel (ClC) 5, a transmembrane protein, which was encoded by *CLCN5*, is responsible for the reabsorption of proteins, calcium, minerals, and vitamins in tubules (5). Hence, Dent 1 disease is mainly characterized by low molecular weight proteinuria and hypercalciuria. In Dent disease, the phenotype heterogeneity often makes the accurate diagnosis difficult. Immunosuppressive medications or renal biopsy are usually prescribed for patients.

In this study, we described a Chinese pedigree with dent disease, in which patients presented asymptomatically with massive proteinuria and hypercalciuria without other diagnostic criteria. Genetic testing was conducted and a novel *CLCN5* delete mutation, which had not been reported previously, was identified in exon 11. This finding expanded the knowledge on the biological role of *CLCN5*. The main clinical features of this disease were also analyzed.

## Cases report

**Figure 1:**
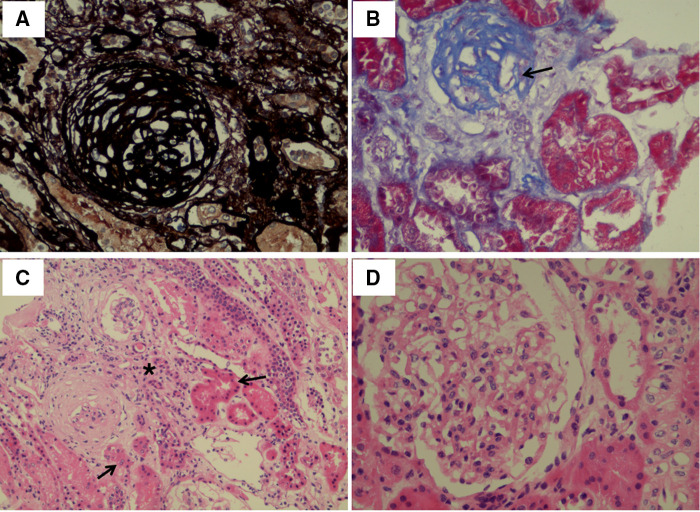
Light microscopy images of kidney tissue. (**A**) Globally sclerostic glomeruli. Silver ×400. (**B**) Globally sclerotic glomeruli (arrow). Masson Trichrome ×400. (**C**) Tubular atrophy (arrow) and renal interstitial fibrosis (star). Hematoxylin-eosin ×200. (**D**) Proliferation of mesangial cells and segmental accumulation of matrix. Hematoxylin-eosin ×400.

Proband, male, 11 years old, was referred to our department for investigation of proteinuria. He was born after a full-term uneventful pregnancy with a birth weight of 4500 g to nonconsanguineous Chinese parents. His mother's uncle (the proband's grandmother's younger brother) was diagnosed with stage 5 chronic kidney disease at the age of 50 years of unknown cause and undergoing dialysis. At the age of 7 years, he had proteinuria (protein in urine dipstick: 2+), but no gross hematuria, no edema and oliguria, no rash and no special treatment was given. After admission, the vital signs, blood pressure, and physical examination all appeared normal. When admitted, the patient was in the 75th and 90th percentiles for weight and height. The serum albumin and cholesterol were normal, while the 24-h urine protein quantification was 2.11 g (55 mg/kg). A high component of LMWP and elevated β-2 microglobulin values up to 54.2 mg/L (normal < 0.2) were revealed by further analysis. These suggested defects in tubules. The 24-h urine calcium excretion was also increased (10 mg/Kg/day) and calcium-to-creatinine ratio was up to 0.48 (normal < 0.21). Serum calcium, potassium, phosphate, complement C3/C4, liver and renal function were normal (serum creatinine 40 umol/L). Autoimmune related antibodies including antinuclear and anti-DNA antibodies were negative. Renal ultrasonography showed increased echogenicity in both renal parenchyma without nephrocalcinosis. Due to the massive proteinuria, kidney biopsy was performed which revealed focal global glomerulosclerosis. Representative findings were displayed in Figure 1. Among the 22 obtained glomeruli from the renal biopsy, two were found to be sclerosed globally. The tubular damage including tubular atrophy and focal interstitial fibrosis were also identified. In addition, there was a mild to moderate proliferation of mesangial cells and segmental accumulation of matrix. By immunofluorescence microscopy, no significant reactivity was detected for immunoglobulin G (IgG), IgM, IgA, or C3 (data not shown). In the mesangial region and the basement membrane, no electron-dense deposits were observed (data not shown).

**Figure 2:**
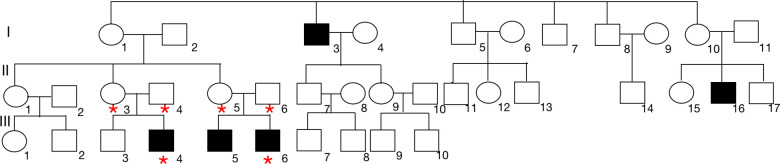
Pedigree analysis of this family. We identified 2 brothers, the proband (III-4) and the affected brother (III-6) who were admitted to our hospital, with proteinuria. The proband was diagnosed with FSGS based on kidney biopsy. The closed boxes represent the patients with proteinuria in this family. The pedigree suggested an X-linked pattern of inheritance. Blood was collected from the proband and 5 additional family members indicated by asterisks.

The Proband's cousin, male, 13 years old, was referred to our department also for proteinuria for half one year. He was the second child of nonconsanguineous Chinese parents. In addition, his older brother was hospitalized in an adult hospital for massive proteinuria and his renal biopsy revealed renal tubulopathy. The clinical manifestations of the child were similar to those of the proband. The detail clinical and laboratory information was demonstrated in Table 1. Based on a survey of both affected brothers, we created a pedigree. The pedigree presented in Figure 2 was suggestive of an X-linked mode of inheritance.

**Table 1:** Laboratory information of the proband and his cousin.

	Proband	Cousin	Normal range
Ages of disease onset	7	12	
Serum pH	7.36	7.35	7.35–7.45
Serum BE (mmol/L)	−1.9	1.0	−4–+4
Serum Calcium (mmol/L)	2.24	2.28	2.196–2.556
Serum Creatinine (μmol/L)	40	45	37–93
Urine β_2_-microglobulin (mg/L)	54.2	63.2	<0.2
Urine α1-microglobin (mg/L)	130	120	0–12
Proteinuria (mg/24 h)	2119	2851	
Urine Calcium (mg/24 h)	398	297.6	
Urine Calcium/Creatinine	0.48	0.28	<0.21
Urine glucose	N	N	
Red blood cell	5–10	4–8	<3/HP
Nephrocalcinosis	N	N	
Nephrolithiasis	N	N	
Osteomalacia	N	N	
Kidney biopsy	FSGS	–	

BE, base excess; N, negative; -, no data; HP, high power lens.

## Diagnostic assessment

**Figure 3:**
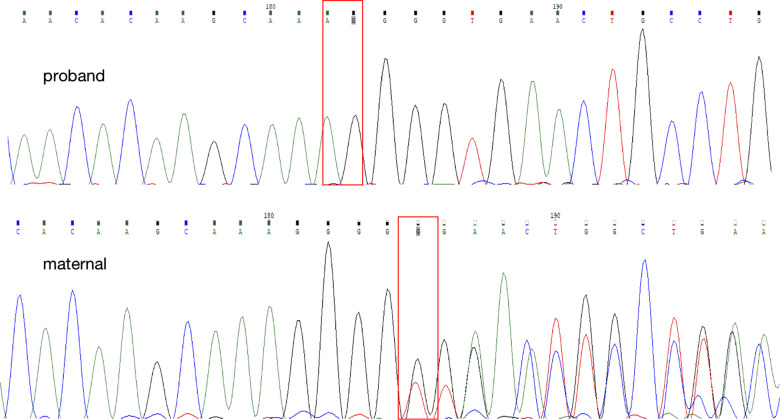
The direct DNA sequencing of the proband and his mother. The red box identified the novel delete mutation (c.1448delG), leading to a single amino acid change at amino acid 483 and to a truncated ClC-5 protein.

Due to family history of renal diseases, persistent LMWP and hypercalciuria, we decided to proceed to genetic testing. Whole exome sequencing was conducted in 6 individuals: the proband, the proband's cousin and their parents. The proband's, his cousin's and their parents' blood were obtained and whole exome sequencing was performed after the informed consent was obtained from each family member. One hemizygous mutation in the DNA sequence of *CLCN5* gene was identified in the mother, c.1448delG in exon 11 (NM_001127899.4) as shown in Figure 3, which resulted in an amino acid mutation: p.Gly483fs. No other pathogenic mutation was detected in either *CLCN5* or *OCRL* gene. This novel mutation was not found in the proband's father, while their mothers were the carrier of this mutation, experienced no clinical evidence of Dent's disease and excreted normal levels of β2-microglobulin in their urine. No previous report of this *CLCN5* mutation has been found in HGMD (http://www.hgmd.org) and ClinVar (http://www.ncbi.nlm.nih.gov/clinvar) nor in the literature before.

## Therapeutic intervention

Both the proband and his cousin are receiving citrate supplementation making urine alkalinization and losartan potassium (1 mg/kg) decreasing proteinuria which is an important factor in the progression of chronic kidney disease (6). At present, one and half years after initial presentation, the proband's renal function is still normal (serum creatinine 41 mmol/L), though accompanied by massive proteinuria (1.63 g, 35 mg/kg) which remains mainly LMWP (β2 microglobulin 126 mg/L, normal < 0.2). Growth is within normal ranges for him. However, his kidneys developed nephrocalcinosis and kidney stones formed, as well as the bone density decreased. The affected cousin has a similar condition as the proband while absence of nephrocalcinosis and low bone density.

## Discussion and conclusions

Our report described Dent disease type 1 with a novel delete mutation of *CLCN5* gene in a Chinese family, characterized by nephrotic range proteinuria, mostly LMWP, and hypercalciuria without any of the other criteria, such as nephrocalcinosis, impaired calcium and bone metabolism at the onset of the disease.

Up to date, it has been reported that there are more than 200 *CLCN5* gene mutations and more than 140 *OCRL* gene mutations associated with Dent disease (7). Ye. et al. analyzed the gene mutation spectrum of Dent disease in 45 Chinese children and revealed 28 different mutations including 17 novel mutations (8). It was found that the mainly *CLCN5* gene mutations were missense (34%) and frameshift (31%) mutations (8, 9). The *CLCN5* gene encodes a voltage-gated chloride/hydrogen ion exchange protein ClC-5, which is expressed in the proximal tubule, the thick ascending limb, and the collecting duct (10). The carboxy terminus of ClC-5 contains two cystathionine B-synthase (CBS) domains which is highly conserved and plays an integral role in chloride/proton exchange and endosomal transport (11). In the present study, this frameshift variant results in premature termination of *CLCN5* protein synthesis, lacking the CBS domains.

The accurate relationship between genotypes and clinical phenotypes has not been discovered. For example, within one family, the phenotype of the same mutation is not identical, with some presenting with severe kidney stones in infancy, while others may only present with mild LWMP or even normal laboratory findings (12, 13). In this case, both the proband and his cousin were absent of nephrocalcinosis at onset, while the proband, not his cousin, developed it during subsequent follow-up. LMWP was found in almost all patients with Dent disease (14). This case was mainly characterized by massive proteinuria with mainly LMWP and hypercalciuria. Additionally, there was no disturbance of calcium and phosphorus metabolism and abnormal renal function. In fact, in the early stage of Dent disease, the loss-of-function mutation of *CLCN5* does not necessarily lead to the occurrence of hypercalciuria or nephrolithiasis, while LMWP is the early and basic manifestation of CLC-5 channel dysfunction (14, 15). Hence, some scholars suggested that if the patient presents with nephrotic level proteinuria and LMWP without edema and hypoalbuminemia, genetic testing should be actively performed to confirm the diagnosis (8, 16). The mechanism whereby these *CLCN5* mutations result in the diverse phenotypes of Dent's disease remains to be elucidated.

The renal biopsy character of type 1 Dent disease is nonspecific, mainly focal segmental glomerulosclerosis (FSGS) or focal global glomerulosclerosis, followed by minimal change disease (MCD) (17). One multicenter analysis of renal biopsy from 30 Dent patients showed that 83% patients had focal global glomerulosclerosis (affecting 16% ± 19% glomeruli) and 70% had renal tubular damage (18). In this case, the presence of focal global glomerulosclerosis (affecting 9.1% glomeruli), was lower than the previous reported data, possibly attributed to the better renal function at biopsy (18).

Clinical management of Dent disease patients is centered on symptoms, rather than on any specific treatment, like most other genetic disorders (19). Hydrochlorothiazide and high citrate diet were used to reduce hypercalciuria in the hope of delaying the progression of chronic kidney disease (20, 21). However, it is necessary to pay attention to the side effects caused by thiazide diuretics, such as severe hypokalemia and dehydration (6). Immunosuppressive agents such as glucocorticoids have no obvious effect. The proband was treated with prednisone and cyclophosphamide at the request of the family before the genetic results were available. The proteinuria was not relieved after the treatment, which was consistent with previous reports (21). It has been reported that angiotensin converting enzyme inhibitors (ACEI) drugs could reduce the urinary protein excretion in patients with type 1 Dent disease (6, 22). On the other hand, Vaisbich et al. did not find benefit from ACEI therapy in patients with type 1 Dent disease (20). In this study, ACEI used by the proband also failed to effectively reduce the urinary protein.

The most serious outcome of Dent disease is renal failure. It was reported that 30% to 80% of male patients can progress to renal insufficiency at the age of 25 to 50 years (23). In this study, neither of the two children showed renal insufficiency. However, long-term follow-up is still needed.

In conclusion, we documented a novel *CLCN5* mutation resulting a frameshift in protein coding sequence, which expanded the spectrum of *CLCN5* mutations. Our understanding of Dent disease heterogeneity is strengthened by this case, which indicates that genetic testing should be done early in cases with high clinical suspicion, despite the absence of all diagnostic criteria. More consideration should be given to the therapeutic options to avoid the undesirable side effects.

## Data Availability

The original contributions presented in the study are included in the article/Supplementary Material, further inquiries can be directed to the corresponding author/s.

## References

[1] Wrong OM Norden AG Feest TG. Dent's disease; a familial proximal renal tubular syndrome with low-molecular-weight proteinuria, hypercalciuria, nephrocalcinosis, metabolic bone disease, progressive renal failure and a marked Male predominance. QJM. (1994) 87:473–93. 10.1093/oxfordjournals.qjmed.a068957 7922301

[2] Jin YY Huang LM Quan XF Mao JH. Dent disease: classification, heterogeneity and diagnosis. World J Pediatr. (2021) 17:52–7. 10.1007/s12519-020-00357-1 32248351

[3] Cho HY Lee BH Choi HJ Ha IS Choi Y Cheong HI. Renal manifestations of dent disease and lowe syndrome. Pediatr Nephrol. (2008) 23:243–9. 10.1007/s00467-007-0686-9 18038239

[4] Bignon Y Alekov A Frachon N Lahuna O Jean-Baptiste Doh-Egueli C Deschenes G A novel CLCN5 pathogenic mutation supports dent disease with Normal endosomal acidification. Hum Mutat. (2018) 39:1139–49. 10.1002/humu.23556 29791050

[5] Scheel O Zdebik AA Lourdel S Jentsch TJ. Voltage-dependent electrogenic chloride/proton exchange by endosomal CLC proteins. Nature. (2005) 436:424–7. 10.1038/nature03860 16034422

[6] Zaniew M Mizerska-Wasiak M Zaluska-Lesniewska I Adamczyk P Kilis-Pstrusinska K Halinski A Dent disease in Poland: what we have learned so far? Int Urol Nephrol. (2017) 49:2005–17. 10.1007/s11255-017-1676-x 28815356

[7] Mansour-Hendili L Blanchard A Le Pottier N Roncelin I Lourdel S Treard C Mutation update of the CLCN5 gene responsible for dent disease 1. Hum Mutat. (2015) 36:743–52. 10.1002/humu.22804 25907713

[8] Ye Q Shen Q Rao J Zhang A Zheng B Liu X Multicenter study of the clinical features and mutation gene spectrum of Chinese children with dent disease. Clin Genet. (2020) 97:407–17. 10.1111/cge.13663 31674016

[9] Gianesello L Del Prete D Ceol M Priante G Calo LA Anglani F. From protein uptake to dent disease: an overview of the CLCN5 gene. Gene. (2020) 747:144662. 10.1016/j.gene.2020.144662 32289351 PMC7402612

[10] Devuyst O Christie PT Courtoy PJ Beauwens R Thakker RV. Intra-renal and subcellular distribution of the human chloride channel, CLC-5, reveals a pathophysiological basis for Dent's Disease. Hum Mol Genet. (1999) 8:247–57. 10.1093/hmg/8.2.247 9931332

[11] Estevez R Pusch M Ferrer-Costa C Orozco M Jentsch TJ. Functional and structural conservation of CBS domains from CLC chloride channels. J Physiol. (2004) 557:363–78. 10.1113/jphysiol.2003.058453 14724190 PMC1665104

[12] Dinour D Davidovitz M Levin-Iaina N Lotan D Cleper R Weissman I Truncating mutations in the chloride/proton ClC-5 antiporter gene in seven Jewish Israeli families with Dent's 1 disease. Nephron Clin Pract. (2009) 112:c262–267. 10.1159/000224793 19546586

[13] Fischer AS Marcussen N Rasmussen M Randers E. Two brothers with identical variants of the CLCN5 gene-one developing Dent's disease. Clin Kidney J. (2018) 11:459–61. 10.1093/ckj/sfx123 30094009 PMC6070071

[14] Sekine T Komoda F Miura K Takita J Shimadzu M Matsuyama T Japanese dent disease has a wider clinical spectrum than dent disease in Europe/USA: genetic and clinical studies of 86 unrelated patients with low-molecular-weight proteinuria. Nephrol Dial Transplant. (2014) 29:376–84. 10.1093/ndt/gft394 24081861

[15] Claverie-Martin F Ramos-Trujillo E Garcia-Nieto V. Dent's disease: clinical features and molecular basis. Pediatr Nephrol. (2011) 26:693–704. 10.1007/s00467-010-1657-0 20936522

[16] van Berkel Y Ludwig M van Wijk JAE Bokenkamp A. Proteinuria in dent disease: a review of the literature. Pediatr Nephrol. (2017) 32:1851–9. 10.1007/s00467-016-3499-x 27757584 PMC5579149

[17] Kubo K Aizawa T Watanabe S Tsugawa K Tsuruga K Ito E Does dent disease remain an underrecognized cause for young boys with focal glomerulosclerosis? Pediatr Int. (2016) 58:747–9. 10.1111/ped.12944 27324082

[18] Wang X Anglani F Beara-Lasic L Mehta AJ Vaughan LE Herrera Hernandez L Glomerular pathology in dent disease and its association with kidney function. Clin J Am Soc Nephrol. (2016) 11:2168–76. 10.2215/CJN.03710416 27697782 PMC5142066

[19] Gianesello L Del Prete D Anglani F Calo LA. Genetics and phenotypic heterogeneity of dent disease: the dark side of the moon. Hum Genet. (2021) 140:401–21. 10.1007/s00439-020-02219-2 32860533 PMC7889681

[20] Vaisbich MH Henriques Ldos S Igarashi T Sekine T Seki G Koch VH. The long-term use of enalapril and hydrochlorothiazide in two novel mutations patients with Dent's disease type 1. J Bras Nefrol. (2012) 34:78–81. 10.1590/S0101-28002012000100013 22441187

[21] Cebotaru V Kaul S Devuyst O Cai H Racusen L Guggino WB High citrate diet delays progression of renal insufficiency in the ClC-5 knockout mouse model of Dent's disease. Kidney Int. (2005) 68:642–52. 10.1111/j.1523-1755.2005.00442.x 16014041

[22] Deng H Zhang Y Xiao H Yao Y Zhang H Liu X Phenotypic spectrum and antialbuminuric response to angiotensin converting enzyme inhibitor and angiotensin receptor blocker therapy in pediatric dent disease. Mol Genet Genomic Med. (2020) 8:e1306. 10.1002/mgg3.1306 32495484 PMC7434612

[23] Anglani F D'Angelo A Bertizzolo LM Tosetto E Ceol M Cremasco D Nephrolithiasis, kidney failure and bone disorders in dent disease patients with and without CLCN5 mutations. Springerplus. (2015) 4:492. 10.1186/s40064-015-1294-y 26389017 PMC4571032

